# Serum microRNAs profile from genome-wide serves as a fingerprint for diagnosis of acute myocardial infarction and angina pectoris

**DOI:** 10.1186/1755-8794-6-16

**Published:** 2013-05-04

**Authors:** Chunjian Li, Zhijuan Fang, Ting Jiang, Qiu Zhang, Chao Liu, Chenyu Zhang, Yang Xiang

**Affiliations:** 1 State Key Laboratory of Pharmaceutical Biotechnology, School of Life Sciences, Nanjing University, 22 Hankou Rd, Nanjing, 210093, China; 2 Department of Cardiology, The First Affiliated Hospital of Nanjing Medical University, Nanjing, China

**Keywords:** Acute myocardial infarction, Angina pectoris, Serum microRNAs

## Abstract

**Background:**

In order to identify miRNAs expression profiling from genome-wide screen for diagnosis of acute myocardial infarction (AMI) and angina pectoris (AP), we investigated the altered profile of serum microRNAs in AMI and AP patients at a relative early stage.

**Methods:**

Serum samples were taken from 117 AMI patients, 182 AP patients and 100 age-and gender-matched controls. An initial screening of miRNAs expression was performed by Solexa sequencing. Differential expression was validated using RT-qPCR in individuals samples, the samples were arranged in a two-phase selection and validation.

**Results:**

The Solexa sequencing results demonstrated marked upregulation of serum miRNAs in AMI patients compared with controls. RT-qPCR analysis identified a profile of six serum miRNAs (miR-1, miR-134, miR-186, miR-208, miR-223 and miR-499) as AMI biomarkers. MiR-208 and miR-499 were elevated higher in AP cases than in AMI cases. The ROC curves indicated a panel of six miRNAs has a great potential to offer sensitive and specific diagnostic tests for AMI. More especially, the panel of six miRNAs presents significantly differences between the AMI and AP cases.

**Conclusions:**

The six-miRNAs signature identified from genome-wide serum miRNA expression profiling may serves as a fingerprint for AMI and AP diagnosis.

## Background

Coronary heart disease (CHD) is a major cause of death in the world’s population. Acute myocardial infarction (AMI) is also a leading source of morbidity and hospitalizations. In US, total CHD prevalence is 7.0% in adults and the overall prevalence for AMI is 3.1%; 18% of coronary attacks are preceded by longstanding angina pectoris (AP) [1]. About 3 million people die from CHD every year and now an estimated 2 million people have AMI in China. In recent years, the survival of AMI patients has improved with better therapeutic strategies such as percutaneous coronary intervention and coronary artery bypass graft [2]. However, the absolute number of deaths has remained almost constant because of an increase in the size of the population and the proportion of older people [3]. An early and correct diagnosis of AMI is critical to enhance the survival of patients.

Now the prediction of AMI is still an urgent matter of attention. Currently, cardiac troponin T (cTnT) and creatine kinase MB (CK-MB) are widely used as the most reliable biomarkers in clinical diagnosis [4]. Elevations in these biomarkers can reflect myocardial necrosis [5]. However, without clinical evidence of ischaemia, we should investigate other aetiologies of myocardial necrosis, such as myocarditis, aortic dissection, pulmonary embolism, congestive heart failure, renal failure [6,7]. Besides, the ECG is an integral part in the diagnosis of suspected AMI [8]. While, ST deviation may be also observed in conditions such as acute pericarditis, LV hypertrophy, Brugada syndrome, and early repolarization patterns [9]. Q-waves may occur due to myocardial fibrosis. Therefore, it is quite essential to explore novel biomarkers with more high sensitivity and specificity for the diagnosis of AMI.

MicroRNAs (miRNAs), endogenous non-coding RNA molecules of 19 to 24 nucleotides in length, are negative regulators of gene expression. We have systematically discovered that miRNAs are stably present in human serum/plasma and circulating miRNAs can serve as biomarkers for various diseases. We have investigated the expression profile of serum miRNA in patients with non-small cell lung carcinoma, colorectal cancer, and type 2 diabetes [10-14]. Recently, miRNAs have been demonstrated to play an important role in AMI. MiR-1, miR-133a, miR-208, and miR-499-5p are considered cardio- or skeletal- muscle specific and are candidates as biomarkers for myocardial infarction [15-20]. However, the global miRNA pattern in the sera of AMI and AP patients has not been determined.

In order to identify miRNAs expression profiling from genome-wide screen for AMI and AP diagnosis, we compared the levels of miRNAs in plasma of 100 control individuals, 117 AMI patients and 182 AP patients in this study. After initial screening by Solexa sequencing, we validated the results at the individual level by using a stem-loop RT-qPCR assay.

## Methods

### Study design, patients and controls

**Figure 1:**
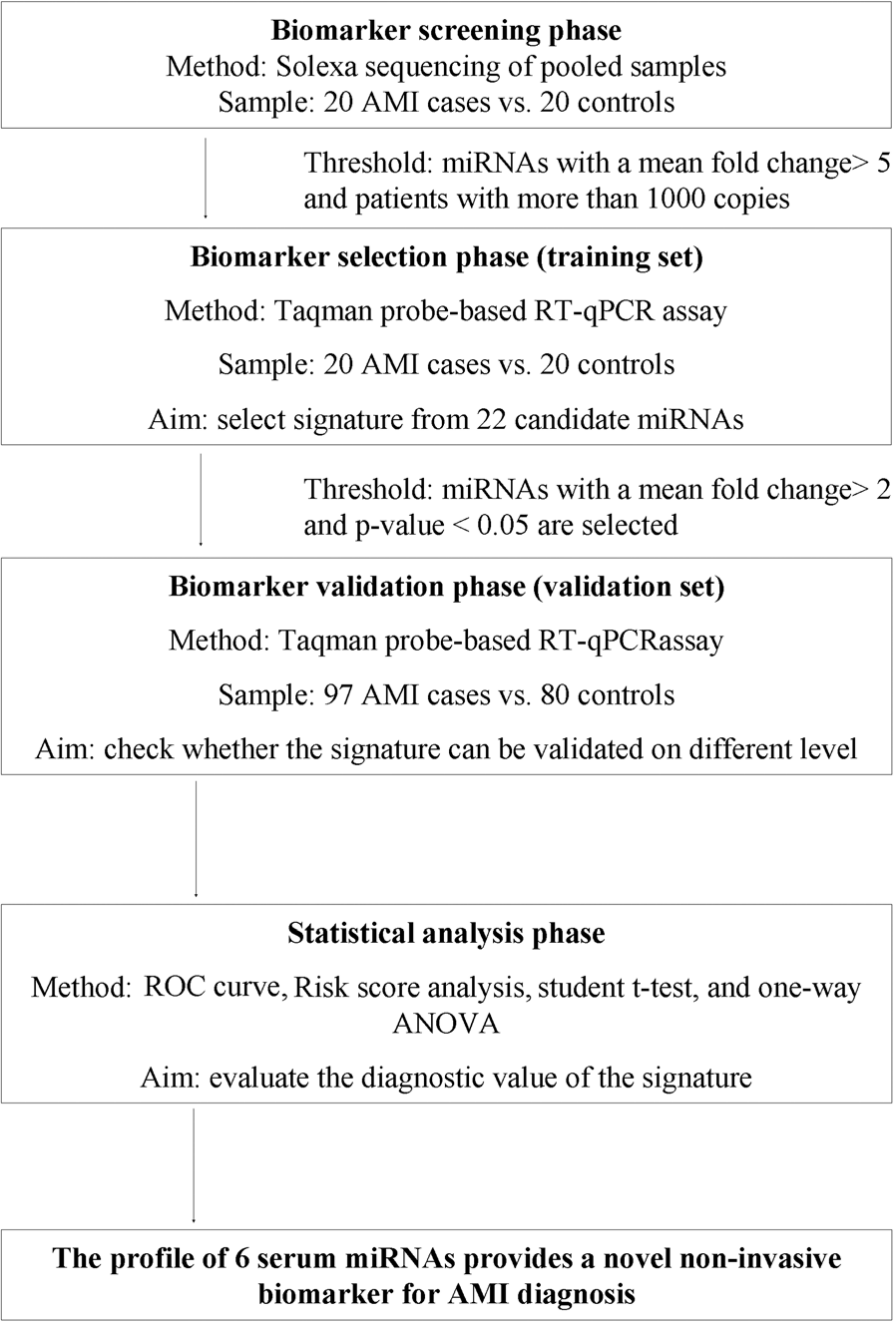
Flow chart of the experimental design.

A multiphase, case–control study was designed to identify serum miRNAs as surrogate markers for AMI (Figure 1). In the initial biomarker-screening stage, pooled serum samples from 20 AMI patients and 20 controls underwent Solexa sequencing (See Additional file 1: Table S1) to identify miRNAs that showed significant differences between the AMI cases and matched controls. Subsequently, we performed a biomarker confirmation analysis with a hydrolysis probe–based RT-qPCR assay to refine the number of serum miRNAs in the AMI signature. This analysis was carried out in 2 phases: (a) the biomarker-selection phase, in which serum samples from 20 AMI patients and 20 control individuals formed the training set, and (b) the biomarker-validation phase, in which serum samples from an additional 97 AMI patients and 80 healthy controls formed the validation set. Besides, the selected miRNAs were also examined by RT-qPCR in the serum samples from 182 AP patients.

Whole blood samples were collected from the Department of Cardiology in the First Affiliated Hospital of Nanjing Medical University between March 2010 and September 2011. The timing of blood collection for the miRNAs analysis is within 2h in the emergency room after hospitalization. The AMI patients were clinically diagnosed as Universal Definition of Myocardial Infarction [21]. The AP patients were clinically diagnosed according to the Guidelines [22]. All patients were eligible according to diagnostic symptoms together with electrocardiogram. Selective coronary arteriography of the right and left coronary arteries was performed on a Digital Cardiovascular X-ray Imaging System. All samples were collected from consenting individuals according to protocols approved by the ethics committee of the First Affiliated Hospital of Nanjing Medical University. The control individuals showed normal electrocardiographic findings and no history of cardiovascular disease. Each participant provided a written informed consent and ethics permission was obtained for the use of blood samples.

### Serum collection and RNA extraction

Separation of the serum was carried out by centrifugation at 3 000 g for 10 min, followed by a 15-min high-speed centrifugation at 12,000 *g* of blood samples. Then, the supernatant sera were stored at −80°C.

For the Solexa sequencing assay, we collected two pools of sera from 20 AMI patients (2 ml each) and 20 healthy controls (2 ml each) separately. The total RNA of each pool was extracted by using Trizol LS Reagent (Invitrogen, Carlsbad, CA) according to the manufacturer’s instructions. Furthermore, we used three steps of phenol/chloroform purification to eliminate remanent proteins.

For the RT-qPCR assay, we extracted total RNA from 200 μl serum through phenol/chloroform purification. Firstly, we mixed 200 μl serum with 200 μl diethylpyrocarbonate-treated water, 200 μl phenol and 200 μl chloroform. Then, we centrifuged the mixture at 12,000 g for 15 min at 25°C, collected the upper aqueous layer and added 40 μl sodium acetate (3mol/L) and 800 μl isopropyl alcohol to it. Subsequently, the solution was put at −20°C for 1 hour. After that, it was centrifuged at 16,000 g for 20 min at 4°C. Then the RNA was washed once by 1 ml 75% ethanol and dried at room temperature. At last the RNA pellet was dissolved in 20 μl diethylpyrocarbonate-treated water and stored at −80°C.

### Solexa sequencing

Firstly, the total RNA was extracted as mentioned above. Through PAGE purification, total small RNA molecules under 30 bp were isolated. After ligating a pair of adaptors to their 5’ and 3’ ends, the small RNA molecules were amplified for 17 cycles and then fragments about 90 bp were isolated from agarose gels. The Illumina Genome Analyzer (Illumina, San Diego, USA) was used for cluster generation and sequencing analysis according to the manufacturer’s instructions. Then we processed the data by computational analysis.

### Quantification of miRNAs by RT-QPCR analysis

Briefly, 2 μl of total RNA was reverse-transcribed to cDNA using AMV reverse transcriptase (TaKaRa, Dalian, China) and the stem-loop RT primer.Real-time RT-qPCR was performed using TaqMan miRNA probes (Applied Biosystems, Foster City, CA, USA) on the Applied Biosystems 7300 Sequence Detection System (Applied Biosystems). All reactions were run in triplicate. After reaction, the threshold cycle (Cq) values were determined using the fixed threshold settings. To calculate the absolute expression levels of the target miRNAs, a series of synthetic miRNA oligonucleotides (dissolved in water) of known concentrations (from 1 fM to 105 fM) were also reverse-transcribed and amplified. The absolute amount of each miRNA was then calculated by referring to the standard curve (See Additional file 1: Figure S1). Since U6 and 5S rRNA are degraded in serum samples and there is no current consensus on housekeeping miRNAs for qRT-PCR analysis of serum miRNAs, the expression levels of miRNAs were directly normalized to serum volume in our study.

### Statistical analysis

Quantitative data are presented as mean ± standard error. Statistical significance was determined using Student’s t-test. P<0.05 was considered statistically significant.

We constructed the ROC curve and calculated the area under the ROC curve (AUC) to evaluate the specificity and sensitivity of AMI and AP prediction. Risk score analysis was also used by us. The risk score of each miRNA denoted as *s*, was set as 1 if the expression level was greater than the upper 95% reference interval for the corresponding miRNA level in controls and as 0 if otherwise. A risk score function (RSF) was defined according to a linear combination of the expression level for each miRNA. The RSF for sample *i* using the information from the six miRNAs was: $RSF_{i}=\Sigma_{j=1}^{6}W_{j}\cdot s_{ij}$.

In the above equation, *s*_*ij*_ is the risk score for miRNA *j* on sample *i* , and W*j* is the weight of the risk score of miRNA *j*. To determine the *Ws*, six univariate logistic regression models were fitted using the disease status with each of the risk scores. The regression coefficient of each risk score was used as the weight to indicate the contribution of each miRNA to the RSF. ROC curves were then used to evaluate the diagnostic effects of the profiling and to find the appropriate cutoff point. All the statistical analyses were performed with Statistical Analysis System software (v.9.1.3; SAS Institute, Cary, NC).

## Results

### Description and clinical features of the patients

In this study, we included 117 AMI patients, 182 AP patients and 100 control individuals. Whole blood samples of patients or healthy donors were collected prior to any therapeutic procedure. The patients were clinically and pathologically diagnosed with AMI or AP at the First Affiliated Hospital of Nanjing Medical University; control participants were also recruited from a large pool of individuals seeking a routine health checkup at the First Affiliated Hospital of Nanjing Medical University. As shown in Table 1, there was no significant difference in the age, gender and ethnicity between the patients and the controls.

**Table 1:** Demographic and clinical characteristics of the patients and control individuals in the training and validation sets

**Characteristics**	**Control** **(n=100)**	**AMI** **(n=117)**	**AP** **(n=182)**	**P**^**1**^	**P**^**2**^	**P**^**3**^
sex (F/M)	19/81	20/97	49/133	7.17×10^-1^	1.97×10^-1^	7.77×10^-2^
age	61.10±7.97	62.70±11.40	65.30±9.98	2.69×10^-1^	4.00×10^-4^	3.47×10^-2^
Hypertension	53/100	69/117	127/182	3.40×10^-1^	4.91×10^-3^	6.81×10^-2^
Diabetes	12/100	29/117	44/182	1.50×10^-2^	1.41×10^-2^	8.72×10^-2^
Glucose	5.37±1.47	6.53±2.79	5.51±1.38	4.47×10^-4^	4.71×10^-1^	7.19×10^-5^
EF%	64.38±5.45	59.19±8.38	63.75±6.67	1.03×10^-5^	4.90×10^-1^	3.41×10^-6^
HDL(mmol/L)	1.17±0.30	1.06±0.28	1.12±0.62	1.84×10^-2^	5.12×10^-1^	4.80×10^-2^
LDL(mmol/L)	2.95 ±0.79	2.81±0.86	2.61±0.77	2.77×10^-1^	8.20×10^-4^	3.57×10^-2^
CK-MB(U/L)	15.26±20.91	80.84±121.17	13.06±12.60	2.10×10^-4^	3.72×10^-1^	6.80×10^-11^
cTnT(ng/ml)	0.15±0.27	0.77±0.74	0.14±0.21	2.77×10^-8^	8.64×10^-1^	6.66×10^-20^

*EF*, Left ventricular ejection fractions; *HDL*, High-density lipoprotein; *LDL*, Low-density lipoprotein; *CK-MB*, Creatine kinase MB; *cTnT*, Cardiac troponin T. *P*^*1*^: comparison between patients with AMI and control. *P*^*2*^: comparison between patients with AP and control. *P*^*3*^: comparison between patients with AMI and AP. *AMI*, acute myocardial infarction. *AP*, angina pectoris.

### Solexa sequencing of serum miRNAs

For the Solexa sequencing assay, we collected two pools of sera from 20 AMI patients (2 ml each) and 20 healthy controls (2 ml each) separately (See Additional file 2: Solexa sequencing). The Solexa data showed that miRNAs were the major components of small RNAs (<30bp) in serum (See Additional file 1: Table S2). Among the 1223 serum miRNAs detected by Solexa sequencing, 394 miRNAs were found in healthy controls and 637 miRNAs were found in AMI patients (See Additional file 1: Table S3). A miRNA was considered altered if Solexa sequencing detected 1000 copies in the patient group and the miRNA showed at least a 5-fold difference between the AMI patients and controls. Based on these criteria, 21 miRNAs showed significant differences between the AMI cases and matched controls (See Additional file 1: Table S4).

### Evaluation of miRNA expression by RT-qPCR

**Figure 2:**
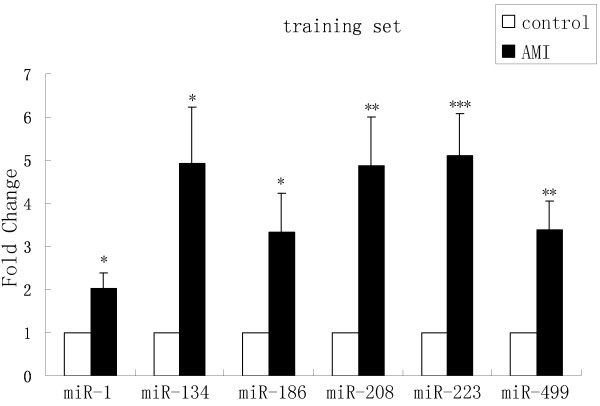
**MiRNAs levels in AMI patients and control samples in the training set.** Serum levels of the six miRNAs were measured in 20 AMI and 20 healthy control subjects using a hydrolysis probe-based RT-qPCR assay. (*P<0.05, **P<0.01, ***P<0.001).

We used RT-qPCR assay to confirm the expression of candidate miRNAs. The RT-qPCR assay for measuring plasma miRNA concentrations was reliable and reproducible (See Additional file 1: Figure S1). MiR-208 was also tested by RT-qPCR because it had been shown to be dysregulated in AMI serum (15). In the training set, miRNAs were measured in a separate set of individual serum samples from 20 AMI patients and 20 healthy controls of the previous step; only miRNAs with a mean fold change > 2 and a p value < 0.05 were selected for further analysis. We used these criteria to generate a list of 6 miRNAs that showed a significant differential expression between AMI patients and controls. Compared to their levels in the control samples, these six miRNAs (miR-1, miR-134, miR-186, miR-208, miR-223 and miR-499) in the AMI samples were increased to 2.02, 4.93, 3.34, 4.87, 5.09 and 3.39 folds, respectively (Figure 2).

**Figure 3:**
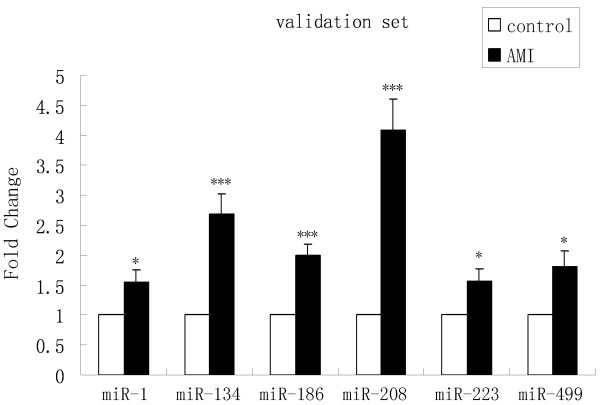
**MiRNAs levels in AMI patients and control subjects in the validation set.** Serum levels of the six miRNAs were measured in 97 AMI and 80 healthy control subjects using a hydrolysis probe-based RT-qPCR assay. (*P<0.05, **P<0.01, ***P<0.001).

**Figure 4:**
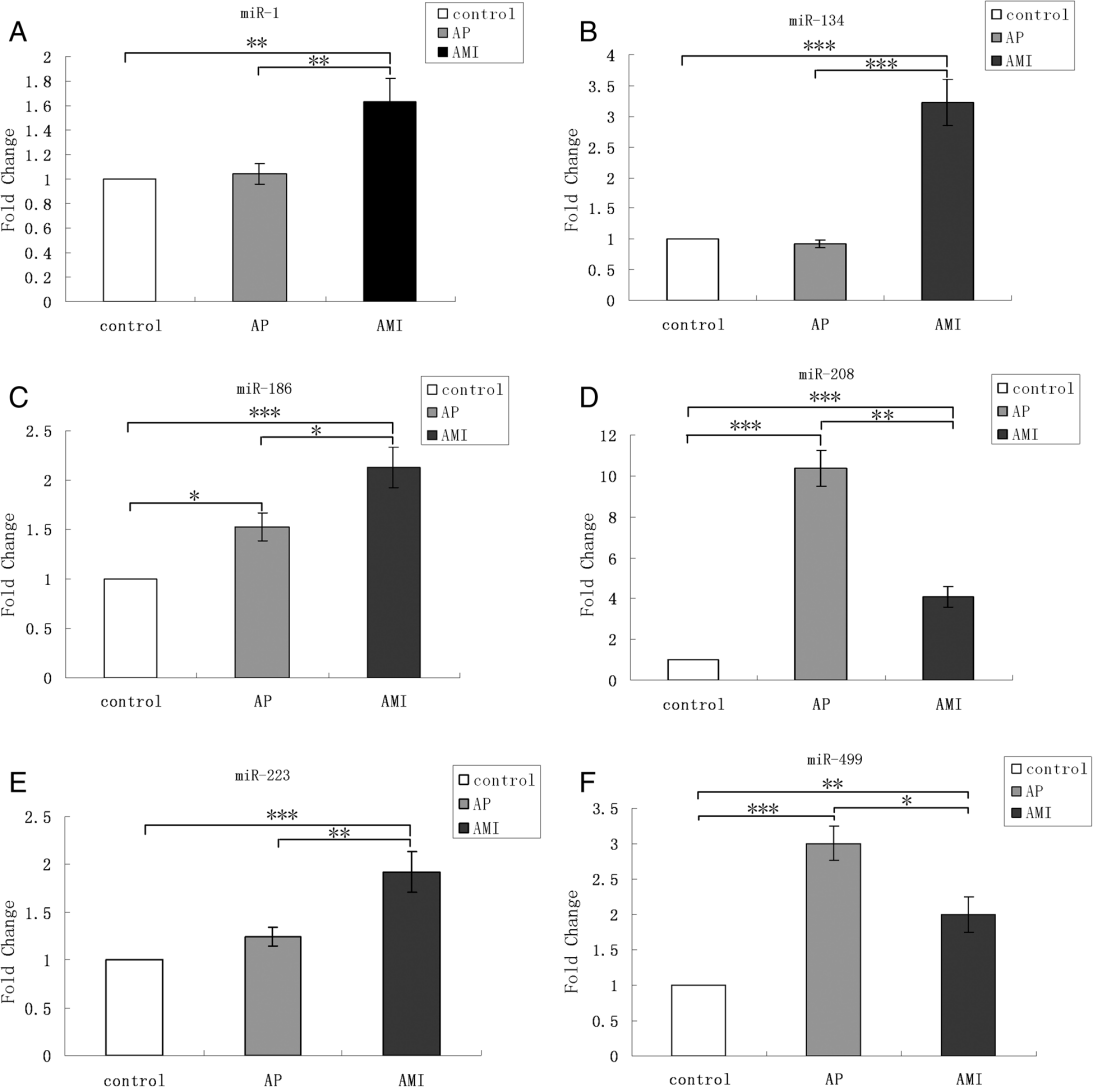
**MiRNAs expression in AMI patients, AP patients and control subjects.** Serum levels of the six miRNAs were measured in 117 AMI cases, 182 AP cases and 100 healthy control subjects using a hydrolysis probe-based RT-qPCR assay (A-F). (*P<0.05, **P<0.01, ***P<0.001).

These six miRNAs were then chosen for the next validation in a larger cohort comprised of 97 AMI patients and 80 matched controls. The miRNAs expression pattern alterations in the validation set were consistent with those in the training set. The levels of the six miRNAs were significantly higher in the AMI cases compared to the control subjects. When compared to their concentrations in normal controls, the fold changes were 1.55, 2.68, 1.99, 4.09, 1.57 and 1.80, respectively (Figure 3). The differential expression of the six miRNAs in the 117 AMI samples compared to the 100 controls is shown in Figure 4A–F.

The selected six miRNAs were also examined by RT-qPCR in the samples from 182 AP patients. The result revealed that three miRNAs (miR-186, miR-208 and miR-499) showed significant difference between the AP patients and controls (Figure 4C-D,F). All these 6 miRNAs presented statistically significant differences between AMI and AP cases, about 1.4 to 3.5 fold changes (Figure 4A-F). Among these 6 miRNAs, miR-208 and miR-499 were elevated higher in AP cases than in AMI cases.

### ROC curve analysis

ROC (receiver-operating characteristic) curves are based on different ways of dichotomous, false-positive rate for the horizontal coordinate, sensitivity to ordinate draws a curve. ROC curve and the area under the curve (AUCs,the area under the curves) can be used as a diagnostic method for evaluation of the accuracy of the indicators. ROC curves constructed to compare the relative concentrations of the 6 miRNAs for AMI patients and healthy controls yielded the following AUCs: miR-1, 0.696 (95% CI, 0.593–0.799); miR-134, 0.657 (95% CI, 0.551–0.763); miR-186, 0.715 (95% CI, 0.614–0.817); miR-208, 0.778 (95% CI, 0.686–0.869); miR-223, 0.741 (95% CI, 0.645–0.838) and miR-499, 0.755 (95% CI, 0.662–0.849) (See Additional file 1: Figure S2, Additional file 1: Table S5). Also, we obtained the AUCs for cTnT and CK-MB and they were 0.800 (95% CI, 0.714–0.887) and 0.683 (95% CI, 0.579–0.786) respectively.

To evaluate the usefulness of the six miRNAs for detecting AP, we performed ROC curve analyses on the selected miRNAs and obtained the respective AUCs (See Additional file 1: Figure S3, Additional file 1: Table S5). Besides, we also calculated that AUCs ranged from 0.591 to 0.764 for the AMI and AP groups (See Additional file 1: Figure S4, Additional file 1: Table S5).

### Risk score analysis

**Figure 5:**
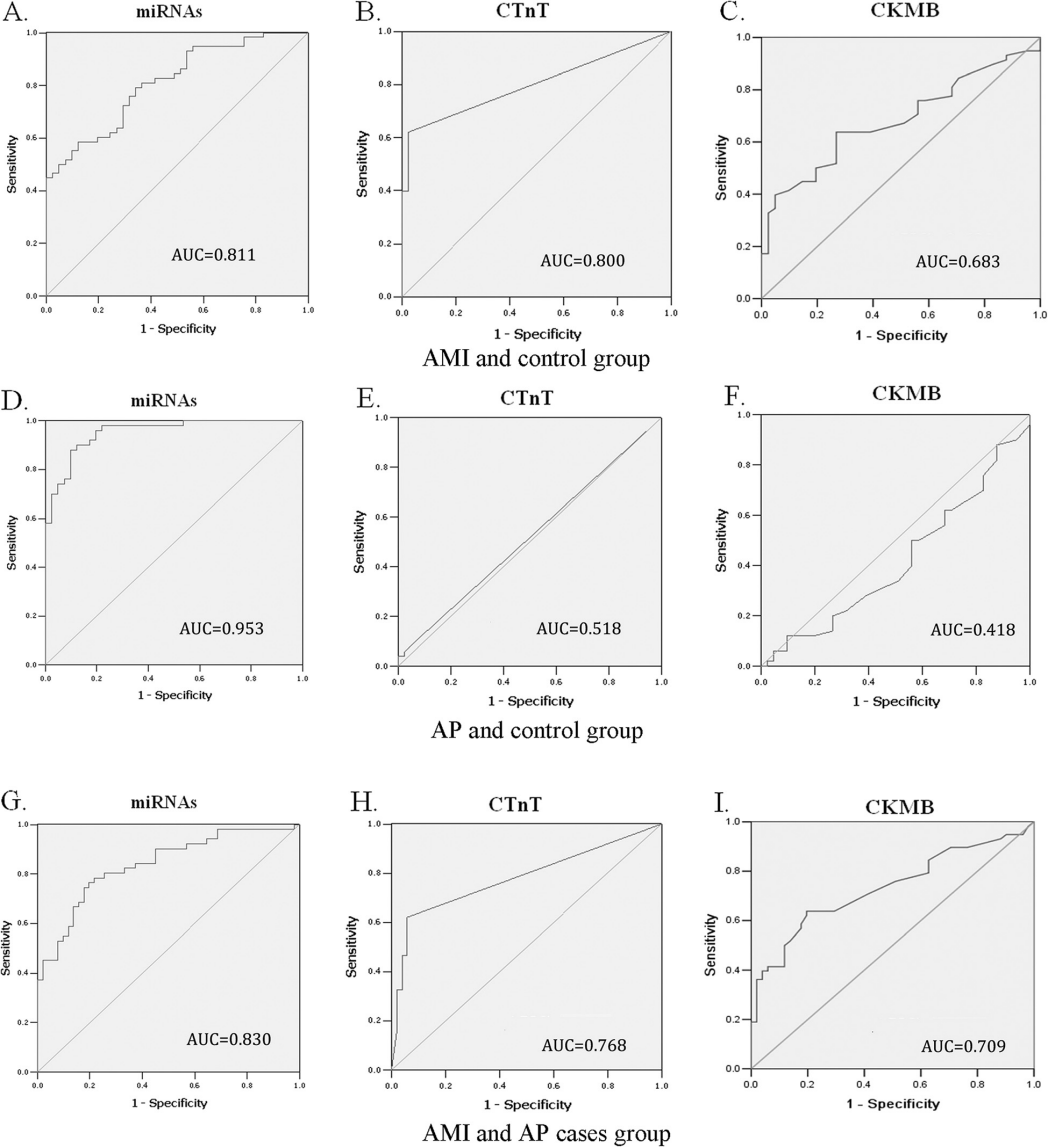
**ROC curves constructed to compare the relative concentrations of the 6 miRNAs.** ROC curves for the 6-miRNAs panel, cTnT and CK-MB to differentiate AMI cases from the controls(**A-C**); ROC curves for the 6-miRNAs panel, cTnT and CK-MB (**D-F**) to differentiate AP cases from the controls; ROC curves for the 6-miRNAs panel , cTnT and CK-MB (**G-I**) to differentiate AMI cases from AP cases.

To further evaluate the diagnostic value of the 6-miRNA profiling system, we used a risk score formula to calculate the RSF for AMI and control samples. Samples were ranked according to their RSF and then divided into a high-risk group representing the predicted AMI cases, and a low-risk group representing the control individuals (See Additional file 1: Table S6). The ROC curves were then used to evaluate the diagnostic effects and to find the appropriate cutoff point. Figure 5A-C shows that the AUC for the 6-miRNAs profiling system was 0.811(95% CI,0.729–0.893). Our results demonstrated that the combination of six miRNAs has a great potential to offer more sensitive and specific diagnostic tests.

We also calculated the RSF for AP and control samples (See Additional file 1: Table S6). Figure 5D-F shows that the AUC value of the 6-miRNAs profiling system was 0.953 (95% CI, 0.915-0.992) markedly higher than those of cTnT (AUC, 0.518; 95% CI, 0.399-0.637) and CK-MB (AUC, 0.418; 95% CI, 0.299-0.536). Expression level of the six miRNAs was also analyzed by risk score for AMI and AP patients. As shown in Figure 5G-I, the AUC values of the six-serum miRNAs signature (AUC, 0.830; 95% CI, 0.751-0.910) were higher than those of cTnT (AUC, 0.768; 95% CI, 0.672-0.864) and CK-MB (AUC, 0.709; 95% CI, 0.606-0.812). The results clearly indicate that a panel of miRNAs has a great potential for AP diagnosis.

## Discussion

As AMI is among the most frequent causes of illness and death, an early diagnosis is essential. Although current studies have revealed a link between the expression of miRNAs and the development of AMI, these studies mainly focused on miRNAs expressed in tissues [17,23,24]. Collecting tissue sample is invasive as it relies on surgical sections. In contrast, serum sample can be obtained with easy accessibility and handled with low cost. Our group has confirmed that miRNAs in human serum and plasma are quiet stable [11,14]. One possible reason is that serum miRNAs are packaged and secreted into the blood within the microvesicles, which also named exosomes or sheding vesicles [14,25-27]. MiRNA-argonaute complexes are also the stable forms in which miRNAs exist in blood [28]. In addition, they may be chemically modified, e.g. methylation [29].

In this study, we performed a high-throughput Solexa sequence assay as an initial screening stage and used multiple RT-qPCR assays with individual serum samples to confirm the serum miRNAs profile. We systematically determined the expression levels of serum miRNAs in AMI patients and identified six serum miRNAs (miR-1, miR-134, miR-186, miR-208, miR-223 and miR-499) significantly up-regulated in AMI patients compared to control subjects. The AUC value based on the six-serum miRNAs signature was progressively higher than any single miRNA-based assay in the diagnosis of AMI. ROC analysis showed that the six-miRNAs profiling system will be a potential complementarity of biomarkers such as cTnT, CK-MB, *etc*. for the clinical diagnosis of AMI.

AP is a common presenting symptom among patients with coronary artery disease, and 6 to 8 percent of patients with AP have myocardial infarction or die within the first year after diagnosis [30]. Our study revealed that miR-186, miR-208 and miR-499 showed significant differences between AP patients and controls. All the 6 miRNAs presented statistically significant differences between the AMI and AP; and risk score based on the six-serum miRNAs signature was progressively higher than the conventional clinical biomarker cTnT and CK-MB. Besides, miR-208 and miR-499 were elevated higher in AP than in AMI cases, which suggests that the two miRNAs may have a higher sensitivity in diagnosis of AP. Although the underlying mechanisms remain unclear, the packaging of specific miRNA populations into microvesicles appears to be a selective process. As a new type of signaling molecule, secreted miRNAs can have biological effects close by or at a distance, can be delivered independent of cell contact or adhesion, and can deliver multiple messages at once and regulate numerous target genes simultaneously, allowing immediate control over target cells [31,32].

The latest results of our group have proved that microvesicles derived from cultured THP-1 cells contain miRNAs and THP-1 cells can selectively package miRNAs into microvesicles in circulating and cultured cell medium under various stimuli including LPS, AGEs, OA/PA and other classic acute or chronic inflammatory factors [33]. We will focus on their critical contribution to pathological processes of coronary heart disease. A better known of the properties and functions of these small regulators of gene expression may favour the design of novel therapeutic approaches for prevention and treatment of coronary heart disease in the future. Here in this study, our results showed that detecting the profile of serum miRNAs may distinguish AMI from AP. Like many other novel biomarkers at their early stages of research, circulating miRNAs require extensive investigation to validate their great potential.

Of the 6 significantly altered miRNAs in AMI, some have been reported to be involved in cardiac injury or protection by altering key signaling elements. It has been shown that miR-499 is involved in inhibiting apoptosis and myocardial infarction induced by anoxia and ischemia through mechanisms involving p53, calcineurin and Drp1 in executing apoptosis program in the heart [34]. MiR-1 is preferentially expressed in adult cardiomyocytes and skeletal muscle, and is known to regulate cardiomyocyte apoptosis through different mechanisms [35-38]. MiR-208 has also been shown to affect muscle function and performance by regulating myosin gene expression [39]. Knockout of miRNA-208 reduced cardiomyocyte hypertrophy and fibrosis in a murine aortic banding model [40]. All of these findings support a role for the selected miRNAs in cardiac function.

## Conclusions

Our results suggested that a combination of six serum miRNAs is more reliable than the single miRNA-based assay in the diagnosis of AMI. A panel of 6-serum miRNAs also is a sensitive indicator of AMI and AP at a relative early stage. Their usefulness in the establishment of a rapid and accurate diagnosis of AMI remains to be determined in unselected populations of patients with acute chest pain.

## Abbreviations

CHD: Coronary heart disease; AMI: Acute myocardial infarction; AP: Angina pectoris; cTnT: Cardiac troponin T; CK-MB: Creatine kinase MB; miRNA: MicroRNA; RT-qPCR: Quantitative reverse transcription PCR; Cq: Threshold cycle; ROC: Receiver-operating characteristic; AUCs: Areas under the curves; RSF: Risk score function; 95%CI: 95% Confidence interval; EF: Left ventricular ejection fractions; HDL: High-density lipoprotein; LDL: Low-density lipoprotein

## Competing interests

The authors declare that they have no competing interests.

## Authors’ contributions

All authors confirmed they have contributed to the intellectual content of this paper and have met the following 3 requirements: (a) significant contributions to the conception and design, acquisition of data, or analysis and interpretation of data; (b) drafting or revising the article for intellectual content; and (c) final approval of the published article. All authors read and approved the final manuscript.

## Pre-publication history

The pre-publication history for this paper can be accessed here:

http://www.biomedcentral.com/1755-8794/6/16/prepub

## Supplementary Material

Additional file 1 Supplementary Table S1-S6 and Figure S1-S4. Click here for file

Additional file 2 Solexa sequencing. Click here for file
