# Supplementary material for: Serum microRNAs profile from genome-wide serves as a fingerprint for diagnosis of acute myocardial infarction and angina pectoris
Source: BMC Med Genomics. 2013 May 4;6:16. doi: 10.1186/1755-8794-6-16 (PMC3655858; doi:10.1186/1755-8794-6-16)
Supplement: Additional file 1 — Supplementary Table S1-S6 and Figure S1-S4. [file 1755-8794-6-16-S1.doc]

**Supplemental Data Tables**

**Supplemental Data Table 1**

The information of AMI patients and healthy controls in Solexa sequencing set.

| Variables | controls | AMI | p value |
| --- | --- | --- | --- |
|  | (n=20) | (n=20) |  |
| sex(F/M) | 9/11 | 5/15 | 0.20 |
| age | 58.2±13.15 | 63.45±6.85 | 0.12 |
| Hypertension | 13 | 13 | 1.00 |
| Diabetes | 3 | 6 | 0.27 |

**Supplemental Data Table 2**

The categories of small RNAs in pooled serum from healthy controls and AMI patients by Solexa sequencing technology.

|  | Controls  (30 ml) | AMI  (30 ml) |
| --- | --- | --- |
| Total  (match genome) | 11259283 | 9364384 |
| rRNA | 6.68% | 28.20% |
| miRNA | 68.92% | 45.02% |
| repeat | 0.40% | 0.71% |
| exon-sense | 0.49% | 0.38% |
| exon-antisense | 0.01% | 0.02% |
| intron-sense | 0.20% | 0.28% |
| intron-antisense | 0.03% | 0.12% |
| srpRNA | 0.00% | 0.00% |
| snRNA | 1.61% | 0.81% |
| piRNA | 0.00% | 0.01% |
| snoRNA | 0.08% | 0.35% |
| scRNA | 0.12% | 0.33% |
| tRNA | 2.15% | 5.40% |
| unannotated | 19.31% | 18.37% |

**Supplemental Data Table 3**

The number of miRNAs in pooled sera from healthy controls and AMI patients by Solexa sequencing technology.

|  | Controls | AMI |
| --- | --- | --- |
| rRNA | 6980 | 6912 |
| miRNA | 394 | 637 |
| repeat | 603 | 742 |
| exon-sense | 344 | 267 |
| exon-antisense | 18 | 19 |
| intron-sense | 201 | 220 |
| intron-antisense | 54 | 91 |
| srpRNA | 1 | 5 |
| snRNA | 635 | 510 |
| piRNA | 3 | 6 |
| snoRNA | 55 | 197 |
| scRNA | 214 | 308 |
| tRNA | 1882 | 2936 |
| unannotated | 35376 | 31324 |

**Supplemental Data Table 4**

Differentially-expressed miRNAs in AMI serum samples compared to controls determined by Solexa sequencing.

| miRNA | Copy number in controls | Copy number in AMI | AMI/controls |
| --- | --- | --- | --- |
|
| hsa-miR-1 | 26036 | 79236 | 3.04 |
| hsa-miR-106b | 0 | 767 | ∞ |
| hsa-miR-125b | 0 | 1742 | ∞ |
| hsa-miR-133a | 0 | 1870 | ∞ |
| hsa-miR-133b | 0 | 761 | ∞ |
| hsa-miR-134 | 0 | 1234 | ∞ |
| hsa-miR-143 | 541 | 21346 | 39.46 |
| hsa-miR-146b-5p | 147 | 4902 | 33.35 |
| hsa-miR-150 | 4 | 7376 | 1843.98 |
| hsa-miR-151-3p | 271 | 14259 | 52.62 |
| hsa-miR-183 | 0 | 1273 | ∞ |
| hsa-miR-186 | 0 | 1106 | ∞ |
| hsa-miR-192 | 835 | 29920 | 35.83 |
| hsa-miR-206 | 517 | 4055 | 7.84 |
| hsa-miR-222 | 11 | 2808 | 255.27 |
| hsa-miR-223 | 265 | 20689 | 78.07 |
| hsa-miR-23b | 50 | 2554 | 51.08 |
| hsa-miR-340 | 0 | 1431 | ∞ |
| hsa-miR-378 | 1336 | 42287 | 31.65 |
| hsa-miR-499-5p | 2 | 15017 | 7508.27 |
| hsa-miR-93 | 0 | 2079 | ∞ |

**Supplemental table 5**

ROC curves and the corresponding AUCs of the six selected miRNAs for the AMI and control groups, for the AP and controls, for the AMI and AP groups.

| Test Result Variable(s) | AMI *vs* Controls | | | | | APs *vs* Controls | | | | | AMI *vs* APs | | | | |
| --- | --- | --- | --- | --- | --- | --- | --- | --- | --- | --- | --- | --- | --- | --- | --- |
|  | Area | Std. Error | Asymptotic  Sig. | Asymptotic  95% Confidence Interval | | Area | Std. Error | Asymptotic  Sig. | Asymptotic  95% Confidence Interval | | Area | Std. Error | Asymptotic Sig. | Asymptotic  95% Confidence Interval | |
|  |  |  |  | Upper Bound | Lower Bound |  |  |  | Upper Bound | Lower Bound |  |  |  | Upper  Bound | Lower Bound |
| miR-1 | 0.696 | 0.053 | 0.001 | 0.593 | 0.799 | 0.640 | 0.058 | 0.022 | 0.527 | 0.753 | 0.591 | 0.058 | 0.116 | 0.478 | 0.704 |
| miR-134 | 0.657 | 0.054 | 0.008 | 0.551 | 0.763 | 0.388 | 0.061 | 0.068 | 0.269 | 0.508 | 0.764 | 0.049 | 0.000 | 0.669 | 0.859 |
| miR-186 | 0.715 | 0.052 | 0.000 | 0.614 | 0.817 | 0.691 | 0.056 | 0.002 | 0.580 | 0.801 | 0.525 | 0.058 | 0.669 | 0.411 | 0.639 |
| miR-208 | 0.778 | 0.047 | 0.000 | 0.686 | 0.869 | 0.905 | 0.029 | 0.000 | 0.847 | 0.962 | 0.674 | 0.054 | 0.003 | 0.568 | 0.779 |
| miR-223 | 0.741 | 0.049 | 0.000 | 0.645 | 0.838 | 0.762 | 0.050 | 0.000 | 0.663 | 0.861 | 0.600 | 0.058 | 0.085 | 0.487 | 0.713 |
| miR-499 | 0.755 | 0.048 | 0.000 | 0.662 | 0.849 | 0.869 | 0.036 | 0.000 | 0.799 | 0.939 | 0.637 | 0.055 | 0.018 | 0.529 | 0.745 |

**Supplemental Data Table 6**

Risk score analysis of AMI cases, AP cases and control subjects on the six-miRNAs profile.

| Score | 0～0.673 | 0.673～1 | PPV*** | NPV** |
| --- | --- | --- | --- | --- |
| Control | 91 | 9 |  | 0.75 |
| AMI | 29 | 88 | 0.91 |  |
| Score | 0～0.545 | 0.545～1 | PPV* | NPV** |
| Control | 94 | 6 |  | 0.75 |
| AP | 31 | 151 | 0.96 |  |
| Score | 0～0.474 | 0.474～1 | PPV*** | NPV** |
| AP | 163 | 19 |  | 0.82 |
| AMI | 37 | 80 | 0.81 |  |

*positive predictive value; **negative predictive value.

**Supplemental Data Figures**

**Supplemental Data Figure 1**

**Supplemental Data Figure 2**

**Supplemental Data Figure 3**

**Supplemental Data Figure 4**

**Supplemental Data Figure legends**

**Figure** **1** Standard curve of miR-1, miR-134, mR-186, miR-208, miR-223 and mir-499 using synthetic miRNAs

**Figure** **2** ROC curve for miR-1, miR-134, mR-186, miR-208, miR-223 and mir-499 to differentiate the AMI cases from the controls

**Figure 3** ROC curve for miR-1, miR-134, mR-186, miR-208, miR-223 and mir-499 to differentiate the AP cases from the controls

**Figure** **4** ROC curve for miR-1, miR-134, mR-186, miR-208, miR-223 and mir-499 to differentiate the AMI cases from the AP cases
